# Med-DGTN: Dynamic Graph Transformer with Adaptive Wavelet Fusion for multi-label medical image classification

**DOI:** 10.3389/fmed.2025.1600736

**Published:** 2025-07-24

**Authors:** Guanyu Zhang, Yan Li, Tingting Wang, Guokun Shi, Li Jin, Zongyun Gu

**Affiliations:** ^1^School of Medical Information Engineering, Anhui University of Chinese Medicine, Hefei, China; ^2^Department of Joint Surgery, Hefei First People’s Hospital, Hefei, China; ^3^Artifcial Intelligence Research Institute of Hefei Comprehensive National Science Center (Anhui Artifcial Intelligence Laboratory), Hefei, China

**Keywords:** Dynamic Graph Transformer, wavelet transform, multi-label classification, medical image analysis, deep learning

## Abstract

**Introduction:**

Multi-label classification of medical imaging data aims to enable simultaneous identification and diagnosis of multiple diseases, delivering comprehensive clinical decision support for complex conditions. Current methodologies demonstrate limitations in capturing disease co-occurrence patterns and preserving subtle pathological signatures. To address these challenges, we propose Med-DGTN, a dynamically integrated framework designed to advance multi-label classification performance in clinical imaging analytics.

**Methods:**

The proposed Med-DGTN (Dynamic Graph Transformer Network with Adaptive Wavelet Fusion) introduces three key innovations: (1) A cross-modal alignment mechanism integrating convolutional visual patterns with graph-based semantic dependencies through conditionally reweighted adjacency matrices; (2) Wavelet-transform-enhanced dense blocks (WTDense) employing multi-frequency decomposition to amplify low-frequency pathological biomarkers; (3) An adaptive fusion architecture optimizing multi-scale feature hierarchies across spatial and spectral domains.

**Results:**

Validated on two public medical imaging benchmarks, Med-DGTN demonstrates superior performance across modalities: (1) Achieving a mean average precision (mAP) of 70.65% on the retinal imaging dataset (MuReD2022), surpassing previous state-of-the-art methods by 2.68 percentage points. (2) On the chest X-ray dataset (ChestXray14), Med-DGTN achieves an average Area Under the Curve (AUC) of 0.841. It outperforms prior state-of-the-art methods in 5 of 14 disease categories.

**Discussion:**

This investigation establishes that joint modeling of dynamic disease correlations and wavelet-optimized feature representation significantly enhances multi-label diagnostic capabilities. Med-DGTN’s architecture demonstrates clinical translatability by revealing disease interaction patterns through interpretable graph structures, potentially informing precision diagnostics in multi-morbidity scenarios.

## 1 Introduction

**Figure 1:**
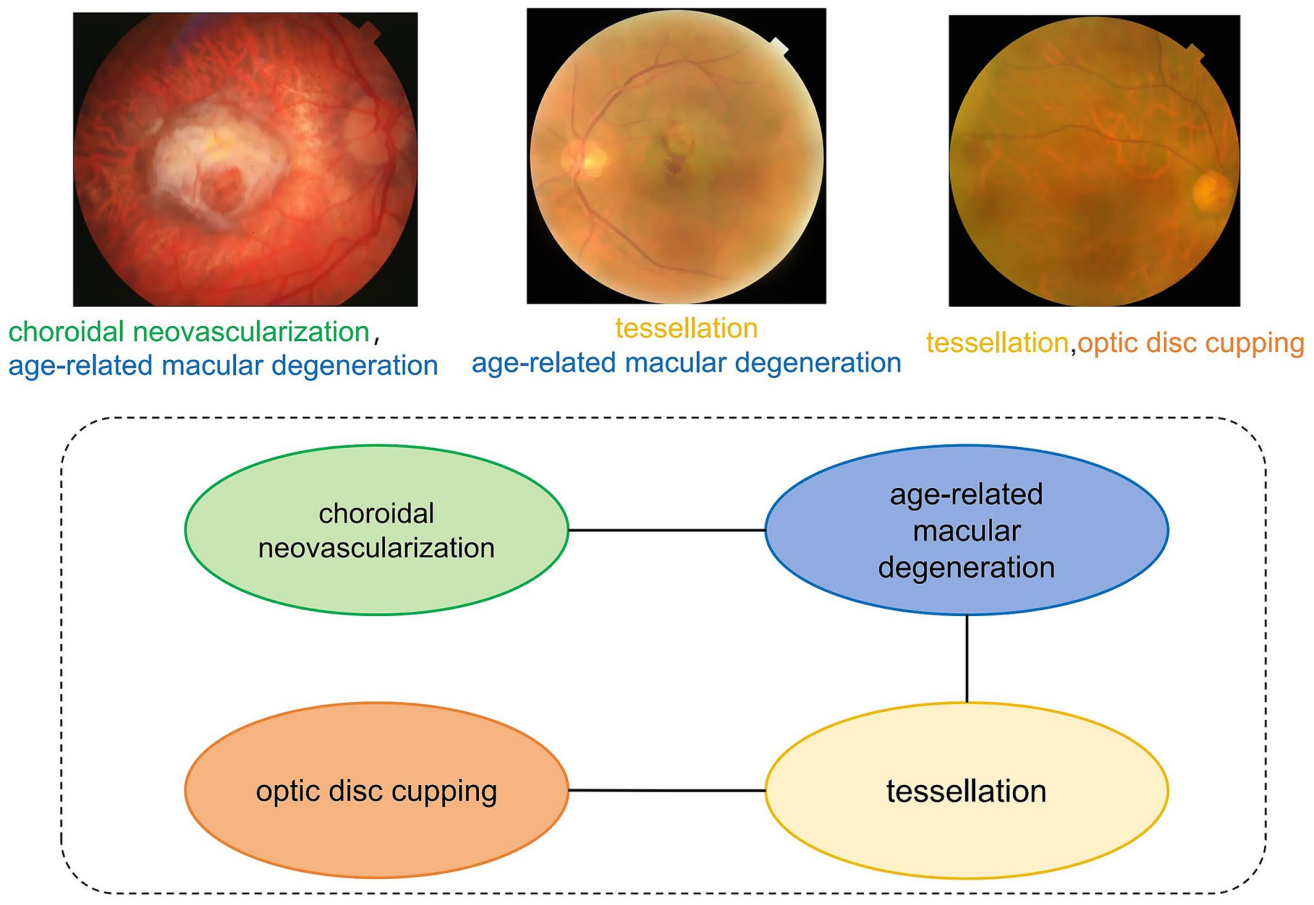
Multi-label dependency diagram of fundus image. This diagram illustrates the co-occurrence relationships among various eye diseases. The labels “choroidal neovascularization” and “age-related macular degeneration” are connected by an edge, indicating a high probability of concurrent occurrence.

The continuous advancement of medical imaging technology has significantly propelled the development of modern precision medicine. The exponential growth of global medical imaging data offers unprecedented opportunities for disease detection and diagnosis (1). However, the co-occurrence of multiple pathologies within individual medical images represents a pervasive challenge across imaging modalities (2). Diabetic retinopathy (DR), a leading global cause of vision loss (3), demonstrates significant clinical comorbidities as evidenced by fundus imaging (Figure 1). Retinal analysis reveals that DR frequently coexists with macular edema (4) and exhibits significant positive correlations with glaucoma progression (5).

Despite the revolutionary impact of deep learning on single-disease detection, multi-label medical image analysis continues to be constrained by three fundamental limitations. Firstly, it overlooks the interdependence of diseases. Conventional binary classification frameworks fail to account for the co-occurrence patterns of diseases, which are particularly critical in progressive conditions like DR, which manifests through stage-specific pathological cascades. Secondly, it suffers from the attenuation of low-frequency features. Early-stage lesions, such as microaneurysms, predominantly reside in low-frequency spectral domains, making them susceptible to information loss in standard Convolutional Neural Network (CNN) architectures (6). Thirdly, it lacks the ability to model dynamic associations. While Graph Convolutional Networks (GCNs) enable the modeling of static relationships, such as those in graph attention networks (7), they fail to adapt to the patient-specific dynamics of disease interactions (8).

We treat a single medical image as a sample, and its multiple diseases as categories, forming a standard multi-label classification problem. This perspective led us to develop the Med-DGTN framework for multi-label classification of medical images. Med-DGTN exhibits enhanced modeling capabilities for pathological associations compared to traditional deep learning-based methods.

The key contributions of this paper include:

- We have developed the Med-DGTN framework, a combination of GCN and CNN. This framework integrates label semantic information with image visual features through a feature space alignment strategy, thereby improving multi-label classification performance.
- We propose a Dynamic Adjacency Matrix Extraction (DAME) module, which initializes from a reweighted correlation matrix based on dataset-level co-occurrence statistics and conditional probabilities. Instead of building a separate graph for each input, we utilize a globally shared label graph whose structure is progressively refined during training via a learnable Graph Transformer. This approach enables adaptive modeling of evolving inter-label dependencies while avoiding the computational cost of per-sample graph construction. As a data-driven strategy for dynamic correlation modeling, it effectively uncovers latent associations among pathological features in medical images.
- We have also designed an image feature extraction module (FEM). This module utilizes the WTDense module, a combination of WTConv layers and multi-scale dense connections. This combination leverages the wavelet transform’s multi-frequency decomposition to enhance the capture of low-frequency pathological features.
- The Med-DGTN framework has demonstrated outstanding performance on public datasets such as MuReD2022 and ChestXray14.

## 2 Related works

In medical image analysis, multi-label image classification presents significant challenges due to the potential for images to display multiple disease characteristics. Accurate identification and categorization of these features are crucial for effective clinical diagnosis. We will now explore the advancements in multi-label medical image classification research.

### 2.1 Multi-label medical image classification

Traditional medical image analysis primarily focuses on diagnosing a single disease, utilizing specialized networks like ConvNext (9) and Vision Transformer (10) for single-label classification. However, multi-disease co-occurrence is prevalent in clinical practice, and single-label methods fall short in modeling label interdependencies.

Researchers have made significant strides to improve multi-label medical image classification. K. V. Priya et al. (11) initiated DenseNet-121 with a pre-trained DenseNet121 from ImageNet, yielding positive results in chest X-ray multi-label tasks. Bingzhi Chen et al. (12) proposed CheXGCN, a GCN-based label co-occurrence learning framework with an Image Feature Embedding (IFE) module and Label Co-occurrence Learning (LCL), significantly enhancing label dependency modeling. Attention mechanisms have also proven beneficial. Li et al. (13) introduced IDSNet, combining DenseNet and SENet modules for high-accuracy breast cancer histopathological image classification. Wu et al. (14) developed CTransCNN, featuring Multi-modality Feature Alignment, Cross-branch Attention, and Interactive Information Mining modules, achieving breakthroughs in multi-label medical image classification.

Despite these advancements, most methods are based on single-label independent prediction assumptions, failing to fully capture dynamic pathological label associations. This limitation restricts their potential in multi-disease co-occurrence analysis. To address these challenges, researchers have introduced GCNs to explore structured label dependencies and have begun integrating frequency-domain information, such as wavelet transforms, to enhance feature robustness and multi-scale representation.

### 2.2 GCNs for medical image applications

In recent years, GCNs have been utilized in medical image processing. Initially, David et al. (15) employed a static graph-based ChebNet for medical image classification, yielding impressive results on the Mayo Clinic cancer disease dataset. However, traditional static graph methods have inherent limitations in modeling complex labels and capturing their dynamic interactions.

To surmount these limitations, You et al. (16) introduced a deep autoregressive model for graph generation, capable of effectively capturing complex joint probabilities of nodes and edges. This advancement facilitated the extraction of node-depth-related features and node classification in graph-structured data of natural images. GCNs (17), by explicitly modeling label topology, have made significant strides in multi-label tasks in natural scenes, inspiring advancements in medical image multi-label classification. Chen et al. (18) improved multi-label classification performance by integrating GCN-learned label features with image features. Yuan et al. (19) further enhanced the model’s expressiveness and adaptability for complex graph-structured data by introducing Graph Transformers.

While GCNs have demonstrated initial success in medical image analysis, current methodologies predominantly remain constrained by static graph modeling paradigms, failing to capture the dynamic interdependencies of pathological labels across temporal disease progression patterns. This fundamental limitation significantly impedes their clinical translatability in patient-specific diagnostic scenarios, urgently necessitating paradigm-shifting innovations to enable dynamic disease association modeling.

### 2.3 Wavelet transform feature enhancement

The Wavelet Transform is increasingly being recognized for its effective multi-scale frequency-domain analysis in image classification. Traditional wavelet-based Convolutional Neural Networks (CNNs) are susceptible to noise interference, leading to skewed results. To enhance noise robustness, Li et al. (20) employed WaveCNets’ frequency-domain decomposition strategy. This approach splits feature maps into low- and high-frequency components for separate processing, achieving high-precision feature extraction and superior noise robustness. Liu et al. (21) utilized the wavelet transform for lesion segmentation, decomposing images to denoise CT scans while preserving lung contours. They combined wavelet transform with fast corner detection to extract pathological details and enhance lung contour correction and segmentation.

This paper addresses the limitations in multi-label medical image classification and inadequate label association modeling. It introduces Med-DGTN, a dual-branch visual-semantic collaborative model. The CNN backbone extracts image features, while the GCN branch, initialized with GloVe word vectors, generates a learnable adjacency matrix through a Graph Transformer layer. Notably, this paper innovatively integrates Discrete Wavelet Transform (DWT) with dynamic graph learning. This combination enhances multi-scale feature extraction using WTConv and a Graph Transformer-driven dynamic topology learning module. This co-optimization of medical image representation and disease semantic associations provides a novel solution for multi-label medical image classification.

## 3 Methodology

**Figure 2:**
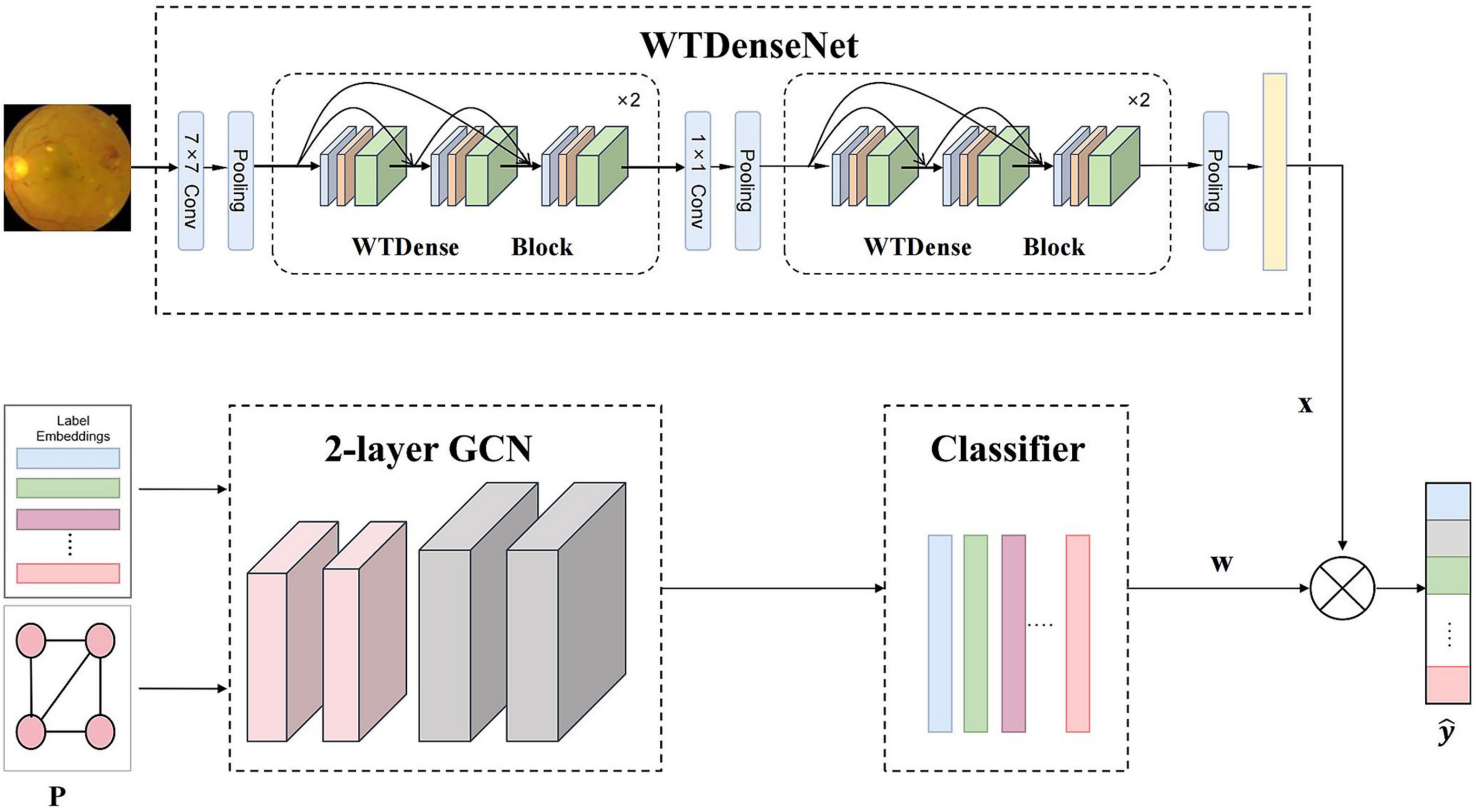
Overall framework of our Med-DGTN model for multi-label medical image classification. The WTDense block is composed of six non-linear combination layers, incorporating batch normalization, ReLU activation, and WTConv. The outputs from these layers are subsequently concatenated across channels. WTDenseNet utilizes cascaded WTDense Block modules as its core structure to extract multi-level image features. These features are subsequently combined with classifiers generated through graph convolution to generate the final predictions.

This study presents the Med-DGTN framework, which augments multi-label classification in medical images by integrating label semantics with visual features. Comprising a Graph Convolutional Network (GCN) module, a dynamic adjacency matrix extractor (DAME), and an image feature extractor module(IFE), the framework facilitates the identification of correlations and dependencies between labels. The dynamic adjacency matrix extractor unveils the evolving relationships among pathological features in multi-label images. Simultaneously, the image feature extractor processes and extracts features from medical images. The components and workflow of the proposed method are illustrated in Figure 2.

### 3.1 Motivations

To address label dependencies, we have developed a dual-branch GCN-CNN framework. The GCN component is responsible for learning and modeling the semantic relationships between disease labels. We initiate the classifier’s label semantic space using GloVe pre-trained word vectors, providing the GCN with initial label association information. Additionally, we have incorporated a Graph Transformer layer into DAME. This layer generates dynamic label correlation matrices that capture dependencies between labels.

Within this framework, the CNN component employs cascaded WTDense Block modules as its backbone to extract multi-level image features. Our focus is on capturing and utilizing correlations between multiple disease labels as prior knowledge to enhance classification performance. In medical image analysis, low-frequency information often contains important pathological features, which are often overlooked in conventional feature extraction methods. Our research indicates that WTConv kernels (22) excel in capturing low-frequency information. Consequently, we have integrated WTConv kernels into WTDense Block modules. This module employs multi-scale feature fusion to improve the accuracy of fine-grained feature extraction, particularly enhancing the processing of low-frequency components in medical images that contain critical pathological information.

### 3.2 GCN module

In the Med-DGTN model designed in this paper, the Graph Convolutional Network (GCN) module passes information between nodes based on the obtained adjacency matrix and updates the node representations. The GCN branch is initialized using GloVe embeddings of disease labels, allowing the model to capture prior semantic relationships, including those involving rare or infrequent labels.

In the model of Med-DGTN constructed in this paper, the GCN (17) module operates by transmitting information between nodes, utilizing the derived adjacency matrix, and consequently updating the node representations.

#### 3.2.1 Graph convolutional network recap

In this study, we employ two stacked GCN layers. Each layer of these networks takes the node representations $H^{l}$ from the preceding layer as input, and subsequently outputs novel node representations $H^{l+1}$ . These are calculated in accordance with Equation 1.


(1) $$H^{\left(l+1\right)}=\sigma ({\tilde{D}}^{-\frac{1}{2}}\tilde{A}{\tilde{D}}^{-\frac{1}{2}}H^{\left(l\right)}W^{\left(l\right)})$$

Here, $\tilde{A}=A+I$ is the adjacency matrix with self-loops added, A is the original adjacency matrix, and I is the identity matrix. $\tilde{D}$ is the degree matrix of $\tilde{A}$ , ${\tilde{D}}_{\mathrm{ii}}=\sum_{j}{\tilde{A}}_{\mathrm{ij}}$ . $W^{\left(l\right)}$ is the learnable weight matrices of the l−th layer. σ is the activation function. The final output of the GCN network is the feature $W\in {\mathbb{R}}^{n\times D}$ , where n is the number of classification labels.

This study employs a mapping function, based on GCN, to develop a classifier W contingent upon the labels (refer to Equation 2).


(2) $$W={\left\{\omega_{i}\right\}}_{i=1}^{C}$$

For the final layer, the output corresponds to the ultimate feature a , with C symbolizing the count of classification labels and D denoting the dimension of the image representation.

#### 3.2.2 Classification label word vector embedding

The input to the GCN network in this module includes the adjacency matrix and the word vector embeddings of the classification label texts. The method for obtaining the adjacency matrix is described in Section 3.3. For label embeddings, we employ 300-dimensional GloVe vectors pretrained. While GloVe is a general-purpose word embedding model, it has shown strong transferability across domains, including biomedical contexts. In particular, many commonly used medical terms such as “glaucoma,” “pneumonia,” and “cardiomegaly” are present and semantically well-captured in the GloVe vocabulary. This allows the model to benefit from meaningful inter-label relational priors at the initialization stage, even before supervised training. The process for embedding the word vectors of the classification label texts is as follows:

First, the classification label texts are preprocessed to obtain clean and standardized input. This involves several steps: the text is first tokenized into individual words, then common stop words are removed, and finally, each word is reduced to its root form through stemming. After these steps, a vocabulary is built by collecting all unique words from the preprocessed text.

Next, a pre-trained word vector model, Glove, is utilized to vectorize each word within the vocabulary. To measure the relationship between words, cosine similarity is calculated between their corresponding vectors, as shown in Equation 3.


(3) $$\text{Similarity}=\cos(v_{i},v_{j})=\frac{v_{i}\cdot v_{j}}{∥v_{i}∥∥v_{j}∥}$$

Here, $V_{i}\in {\mathbb{R}}^{300}$ and $V_{j}$ denote the 300-dimensional Glove embeddings of the i -th and j -th words, respectively. The cosine similarity serves as edge weights in the graph construction.

Finally, the network’s embedding layer maps each word’s index to its corresponding word vector. Throughout the model’s forward propagation, these word vector representations are channeled to subsequent neural network layers for further processing.

### 3.3 DAME module

**Figure 3:**
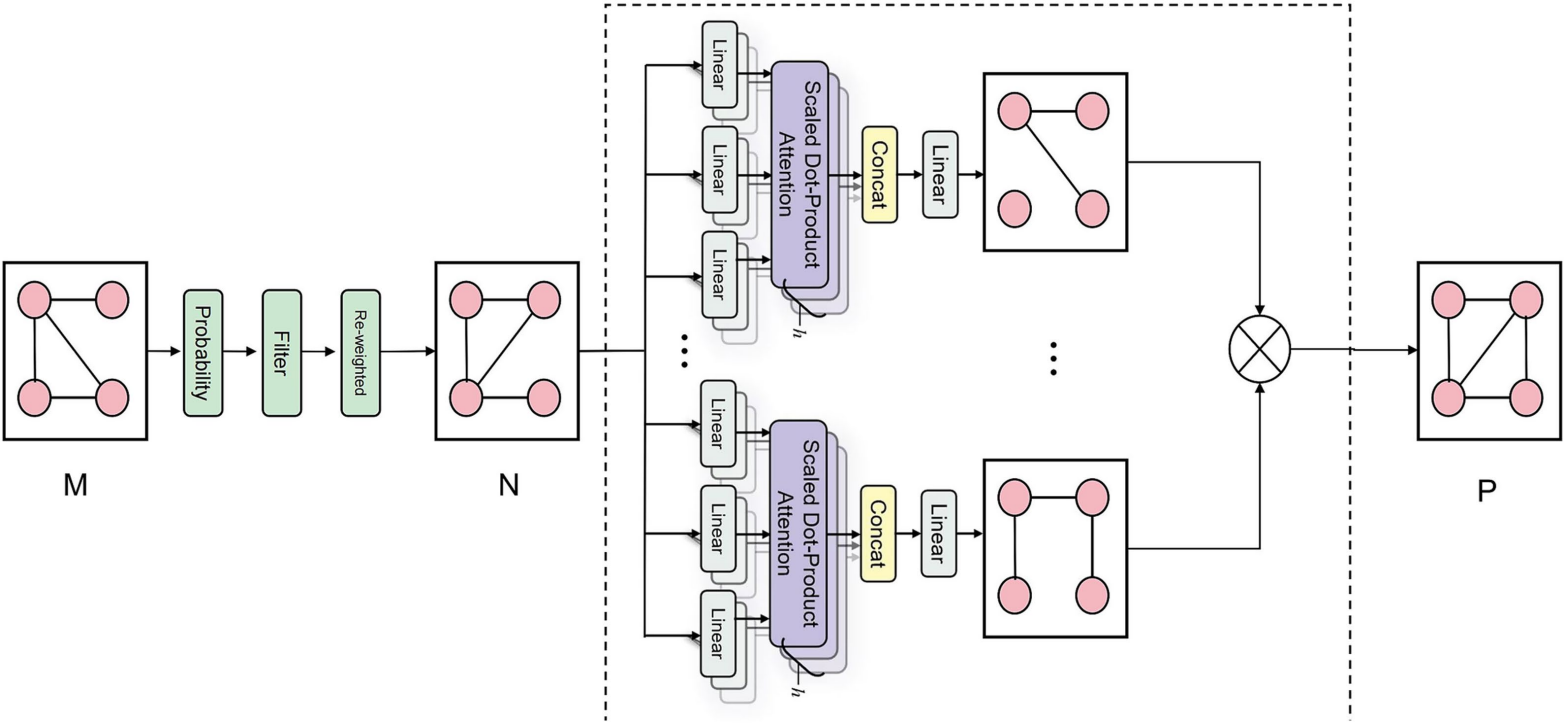
Schematic diagram of DAME module correlation matrix generation. The schematic diagram of the DAME module’s correlation matrix generation illustrates its processing of the co-occurrence matrix M. This process involves conditional probability modeling to quantify disease relationships, followed by noise filtering and reweighting to reduce spurious correlations. Finally, a GAT layer is applied to enhance the capture of structural information related to disease-label nodes, ultimately producing a refined graph structure P with enriched node representations.

The DAME module, a vital component of our framework, excavates dynamic associations among various pathological features from medical image data. As illustrated in Figure 3, the process commences by obtaining a correlation matrix N , from a multi-label medical image dataset. Subsequently, a Graph Transformer Network (GTN) is employed to generate an adjacency matrix, P . The DAME module further reinforces this by constructing a dynamic label graph using co-occurrence statistics and conditional probabilities as initial priors, which are further optimized through a learnable Graph Transformer. Rather than assigning a separate graph for each individual sample, this approach models a globally shared label graph that is dynamically refined throughout training. This enables the model to capture contextual dependencies and strengthens representations for underrepresented classes via shared label semantics.

#### 3.3.1 Computing the correlation matrix

Computing the correlation matrix between disease labels can reveal concealed relationships and provide crucial insights for model design and optimization. The process involves the following steps:

Firstly, we quantify the co-occurrence frequency of various disease label pairs within the training set of multi-label medical image classification, constructing a label co-occurrence matrix M , $M\in {\mathbb{R}}^{C\times C}$ .

Secondly, we model the correlations and dependencies between labels using conditional probability, generating a conditional probability matrix, M′ . To mitigate noise and over-smoothing inherent in simple correlations, we implement a threshold, τ , to eliminate low-confidence edges, thereby creating a binary matrix, A , as illustrated in Equation 4.


(4) $$A_{\mathrm{ij}}=\{\begin{array}{c} 0,\;\;\text{if}\;M_{\mathrm{ij}}^{\prime }<\tau \\ 1,\;\;\text{if}\;M_{\mathrm{ij}}^{\prime }\geq \tau \end{array}$$

Finally, the correlation matrix is derived. To mitigate the issue of over-smoothing inherent in the binary-valued correlation matrix, a reweighting strategy is implemented, as demonstrated in Equation 5.


(5) $$N_{\mathrm{ij}}=\{\begin{array}{c} \frac{p}{\sum_{i=1}^{C}A_{\mathrm{ij}}\;},\;\;\text{if}\;i\neq j\\ 1-p,\;\;\;\;\;\text{if}\;i=j\; \end{array}$$

Here, p is a hyperparameter that balances the importance between self-connections and inter-node connections. When p approaches 1, the model emphasizes relationships between different nodes and downplays the self-connection. Conversely, when p approaches 0, the self-connection dominates, reducing the influence of other nodes.

#### 3.3.2 Adjacency matrix computation

This study introduces a Graph Attention Transformer (GAT) layer to enhance the capture of structural information related to disease label nodes. The previously obtained correlation matrix, N, is transformed into a new graph structure, P. The process involves several steps:

Firstly, the correlation matrix, N, is processed through distinct linear layers to produce the query matrix, Q, the key matrix, K, and the value matrix, V (23), as shown in Equation 6.


(6) $$\left[Q_{i},K_{i},V_{i}]=N[W_{i}^{Q},W_{i}^{K},W_{i}^{V}\right]$$

Here, $W_{i}^{Q},W_{i}^{K},W_{i}^{V}\in {\mathbb{R}}^{n\times D_{h}}$ 。

Secondly, the attention matrix is computed based on Q,K,V , as depicted in Equation 7.


(7) $$\text{Attention}(Q_{i},K_{i},V_{i})=\text{softmax}(\frac{Q_{i}K_{i}^{T}}{\sqrt{D_{h}}})V_{i}$$

Thirdly, for each attention layer head h , a subgraph G containing information related to the disease label nodes is derived through computation, as illustrated in Equation 8.


(8) $$G_{j}=\text{Concat}(\text{Attention}(Q_{1},K_{1},V_{1}),\ldots ,\text{Attention}(Q_{h},K_{h},V_{h}))W^{0}$$

Lastly, the adjacency matrix for the GCN is obtained by performing matrix multiplication on the subgraphs G from all attention heads, as demonstrated in Equation 9.


(9) $$P=\prod_{j=1}^{k}\;G_{j}\;$$

### 3.4 IFE module

**Figure 4:**
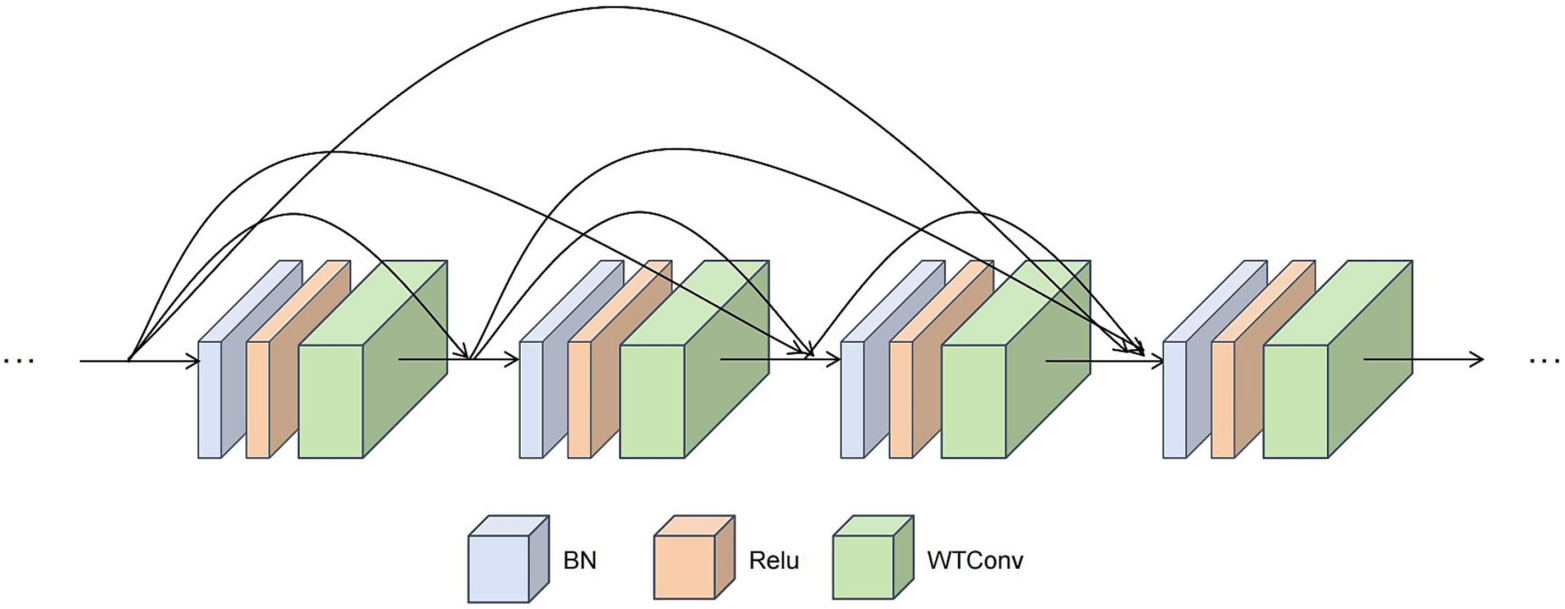
Schematic diagram of The WTDense block module. This module employs a dense connection pattern. Within each WTDense Block, following the application of BN and ReLU, a WTconv operation is conducted. The feature maps generated by each layer are then concatenated along the channel dimension, thereby serving as the input for the subsequent layers.

In the Med-DGTN model, the IFE is a CNN with WTDense Block modules (Figure 4). Each of the six nonlinear combination functions in a WTDense Block includes batch normalization (BN), ReLU activation, and WTConv. Each WTDense Block is followed by a Transition Layer with 1 × 1 convolution and 2 × 2 average pooling. After the last WTDense Block, there is a global average pooling layer.

#### 3.4.1 Image feature extraction

The feature extraction network commences with a 448 × 448 medical image as input.

First, the process initially involves passing the image through an initial visual layer, which comprises a 7 × 7 convolutional layer and a pooling layer. These components extract low-level features.

Next, the data is channeled into the first WTDense Block. Within this block, the data undergoes a series of nonlinear layers. Each nonlinear layer performs the following operations in sequence:

- Applies batch normalization to the input feature map to stabilize the training process and accelerate convergence.
- Applies a ReLU activation function to introduce nonlinearity and enhance the expressive power of features.
- Applies a 3 × 3 WTConv layer for feature extraction based on wavelet convolution.

Each layer’s output is subsequently concatenated with the input feature map via channel-wise concatenation. The WTConv, which integrates cascaded wavelet decomposition, employs small convolutional kernels. With each wavelet transformation level, the receptive field expands, albeit with a slight increase in parameters.

After undergoing processing through multiple WTDense Blocks, the feature map size diminishes to 2048 × 14 × 14. In medical images, low-frequency information frequently contains crucial anatomical and pathological features. The WTConv accentuates these low-frequency components through repeated wavelet decomposition, thereby operating on multiple frequencies with compact kernels. This approach aids the model in better handling noise and inhomogeneity in medical images, thereby enhancing classification robustness. Moreover, these low-frequency features are often associated with rare or subtle disease manifestations. By focusing on both prominent and hidden pathological signals, the model improves its ability to generalize under class-imbalanced conditions and strengthens its practical applicability in real-world clinical settings.

Finally, global average pooling compresses the feature map into a 1D feature $x\in {\mathbb{R}}^{D}$ (D = 2048). This vector is then utilized for feature fusion with the output of the graph convolution operation.

#### 3.4.2 Feature fusion

The integration of the one-dimensional feature, x, extracted from the feature extraction module, with the feature, W, learned by the GCN (refer to Equation 2), enables the generation of prediction scores, $\hat{y}$ , for the multi-label classification task (refer to Equation 10).


(10) $$\hat{y}=\mathrm{Wx}$$

#### 3.4.3 Loss function

Within the Med-DGTN framework, the entire network is trained utilizing a multi-label classification loss function, denoted as ℒ (referenced in Equation 11). Here, $y\in {\mathbb{R}}^{C}$ represents the true labels of the image, $y^{i}=\{0,1\}$ denoting the presence or absence of each label i .


(11) $$ℒ=\sum_{c=1}^{C}y^{c}\log(\sigma ({\hat{y}}^{c}))+(1-y^{c})\log(1-\sigma ({\hat{y}}^{c}))$$

## 4 Experiments

In this section, we first introduce the evaluation metrics and experimental settings. Then, we visualize the generated correlation matrix and present the comparison results of different feature extractors. Next, we test the Med-DGTN model and compare its experimental results with those of current advanced models. Finally, we validate the effectiveness of each module in the model through a series of ablation experiments.

### 4.1 Evaluation metrics

To thoroughly evaluate the performance of the Med-DGTN model in multi-label medical image classification, we utilized evaluation systems specifically designed to suit the characteristics and data distribution of each dataset.

For the MuReD2022 dataset, we utilized common multi-label classification metrics (24). Metrics such as Overall Precision (OP), Overall Recall (OR), and Overall F1 score (OF1) provide a broad overview of the model’s performance across all labels. In contrast, Class-wise Precision (CP), Class-wise Recall (CR), and Class-wise F1 score (CF1) measure the model’s ability to identify individual pathological categories. Furthermore, we incorporated the composite metric, mean Average Precision (mAP), which calculates the average area under the Precision-Recall curve for each label, to assess the model’s robustness in multi-label scenarios. Additionally, we retained the “Other” label to test the model’s recognition of rare diseases.

For the ChestXray14 dataset, given its severe class imbalance, we opted for the Area Under the Curve (AUC) (25) as the evaluation metric. AUC, which computes the area under the curve of the true positive rate versus the false positive rate, effectively mitigates the impact of class distribution skew on evaluation results. This metric is extensively used in multi-label chest X-ray classification tasks (26).

### 4.2 Experimental details

This paper utilizes the PyTorch deep learning framework for development in Python 3.8. The experimental hardware comprises an RTX 4090 GPU, and the software environment consists of PyTorch 2.5.1 and CUDA 12.1. In the image feature extraction module, a CNN backbone with pre-trained weights loaded is integrated to boost model performance.

The training process employs the Stochastic Gradient Descent (SGD) optimizer, setting the momentum at 0.9 and the weight decay coefficient at 10-4. The initial learning rate for the Dynamic Convolutional Graph Network is set at 0.5, while for CNN, it is set at 0.03. The higher initial learning rate aids the network in quick convergence during the early training stages. Later on, reducing the learning rate helps prevent oscillation near the optimal solution, ensuring stable convergence. The entire training process is configured for 100 epochs, with the learning rate decaying to 1/10 of its current value every 30 epochs. The batch size is set to 16. To mitigate overfitting, an Early Stopping mechanism is incorporated into the training strategy.

In the data preprocessing stage, all images are resized to 448×448 and standardized. Data augmentation techniques, such as random horizontal flipping and multi-scale cropping, are also applied, along with normalization, to ensure consistent data distribution across channels. These preprocessing steps improve image quality, significantly enhancing the model’s performance and generalization ability.

The DAME module involves two hyperparameters: the threshold τ used to filter weak connections in the conditional probability matrix and the re-weighting parameter p for balancing self-loops. In our experiments, τ is set to 0.4 and p is set to 0.2, following the configuration used in Chen et al. (18). These values were found to yield stable performance while effectively preserving meaningful label dependencies.

### 4.3 Adjacency matrix heatmaps

**Figure 5:**
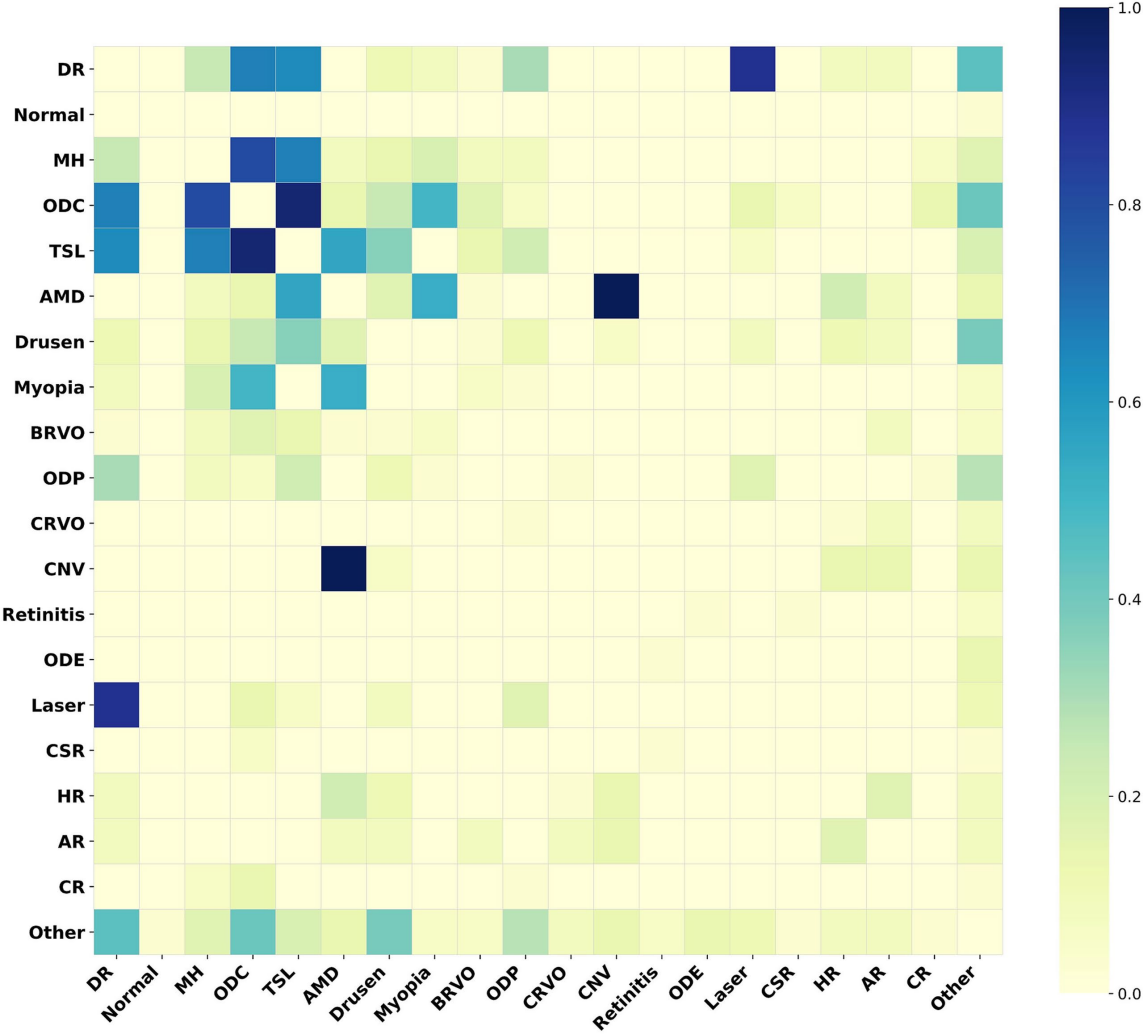
Heatmap of the adjacency matrix for MuReD2022. This heatmap illustrates the correlation strengths between the fundus disease labels within the dataset. The darker the color, the stronger the correlation between the corresponding labels.

For the MuReD2022 retinal disease multi-label dataset, we illustrate the role of the adjacency matrix in the model by plotting a heatmap of correlations among all fundus disease labels. In Figure 5, disease label abbreviations denote twenty labels, including ‘diabetic retinopathy’, ‘normal’, ‘media haze’, ‘optic disc cupping’, ‘tessellation’, ‘age-related macular degeneration’, ‘drusen’, ‘myopia’, ‘branch retinal vein occlusion’, ‘optic disc pallor’, ‘central retinal vein occlusion’, ‘choroidal neovascularization’, ‘retinitis’, ‘optic disc edema’, ‘laser scars’, ‘central serous retinopathy’, ‘hypertensive retinopathy’, ‘arteriosclerotic retinopathy’, ‘chorioretinitis’, and ‘other diseases’.

Figure 5 indicates a strong correlation between ‘age-related macular degeneration’ and ‘choroidal neovascularization’, indicating a high probability of their co-occurrence. Conversely, a weak correlation between these two conditions implies a low probability of co-occurrence. The Med-DGTN model incorporates these correlations, employing conditional probability modeling and reweighting strategies, and utilizes the Graph Transformer Net to generate a correlation matrix that reflects intricate label relationships. This matrix not only aids in guiding the model’s classification training and inference but also serves as a constraint that enhances disease prediction accuracy by influencing multi-label predictions.

**Figure 6:**
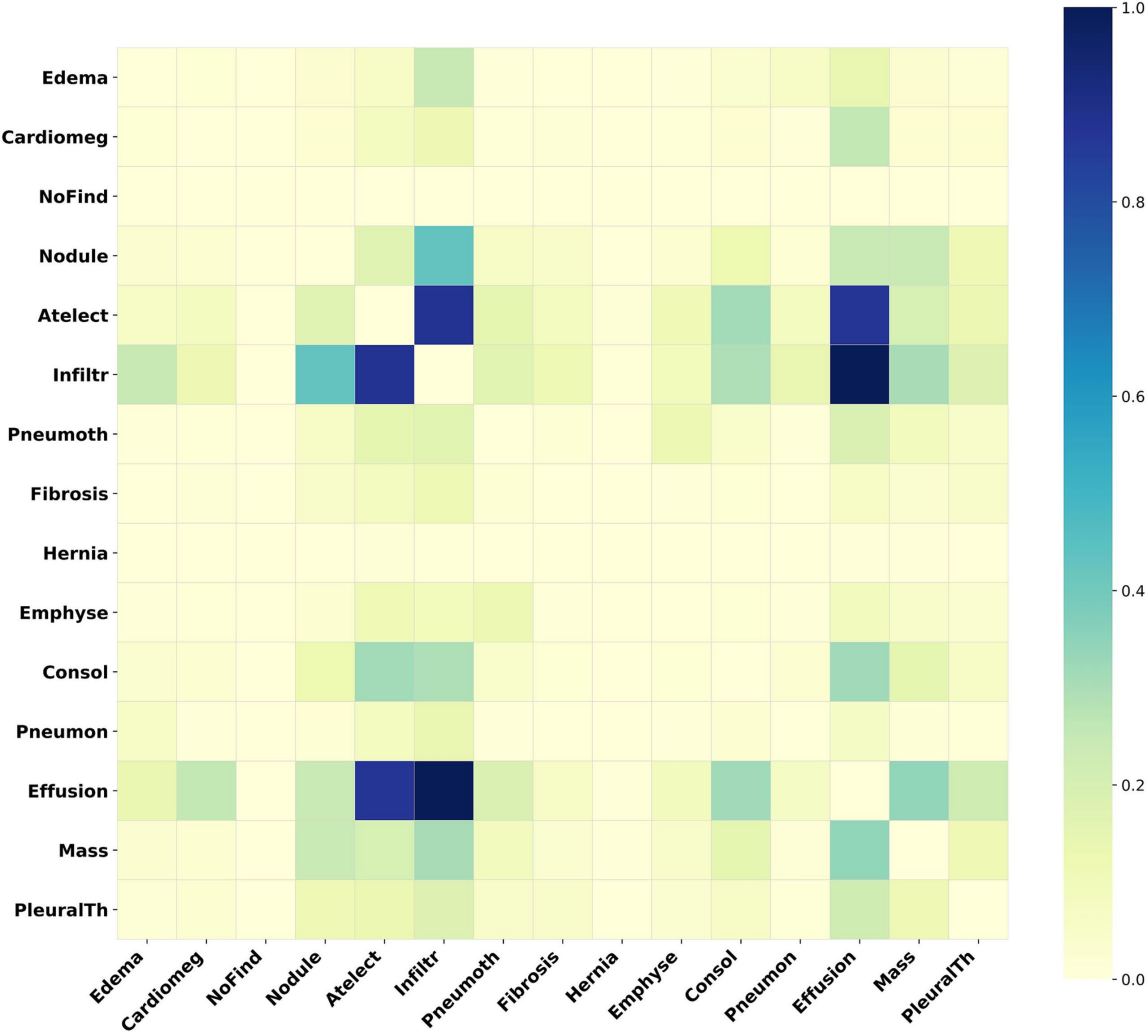
Heatmap of the adjacency matrix for ChestXray14. This heatmap illustrates the inter-label correlation patterns among the various disease labels within this dataset.

For the ChestXray14 dataset, disease label abbreviations in Figure 6 correspond to fifteen labels: “edema,” “cardiomegaly,” “no finding,” “nodule,” “atelectasis,” “infiltration,” “pneumothorax,” “fibrosis,” “hernia,” “emphysema,” “consolidation,” “pneumonia,” “effusion,” “mass,” and “pleural_thickening.” Figure 6 presents a label correlation heatmap, revealing associations between various chest diseases.

### 4.4 Choice of the FEM’s backbone network

To achieve optimal model performance, we compared different image feature extraction networks on the MuReD2022 dataset. All models used the basic network architecture without our enhanced WTDense Block module. Each model had two GCN layers, with consistent configurations and input parameters. Table 1 details the experimental results.

**Table 1:** Comparison of different networks as feature extractors.

Backbone	mAP	OP	OR	OF1	CP	CR	CF1
ResNet-50	57.839	0.7222	0.5601	0.6309	0.5542	0.4601	0.5028
ResNet-101	57.859	0.7186	0.5548	0.6261	0.5943	0.4750	0.5280
ResNeXt-50 32x4d	56.389	0.6757	0.5835	0.6262	0.4688	0.4336	0.4505
ResNeXt-101 32x16d	63.248	0.7386	0.6086	0.6673	0.6459	0.5341	0.5847
VGG16	57.817	**0.7467**	0.5081	0.6047	0.5607	0.3787	0.4521
DenseNet161	**67.960**	0.7137	**0.6625**	**0.6872**	0.6189	**0.5708**	0.5938
ConvNeXt	60.045	0.7338	0.5494	0.6283	0.5843	0.4625	0.5163
Swin Transformer	65.517	0.7248	0.6194	0.6680	**0.6835**	0.5415	**0.6043**

This table compares the key characteristics of various networks serving as feature extractors. The metrics assessed include mAP, OP, OR, OF1, CP, CR, and CF1, serving as a benchmark for the selection of an appropriate feature extraction network. The data suggests that DenseNet161 exhibits the best overall performance.

As shown in Table 1, DenseNet161 performed best among the tested backbone networks. It achieved the highest mAP and outperformed other models in key metrics like OR, OF1, and CR.

**Figure 7:**
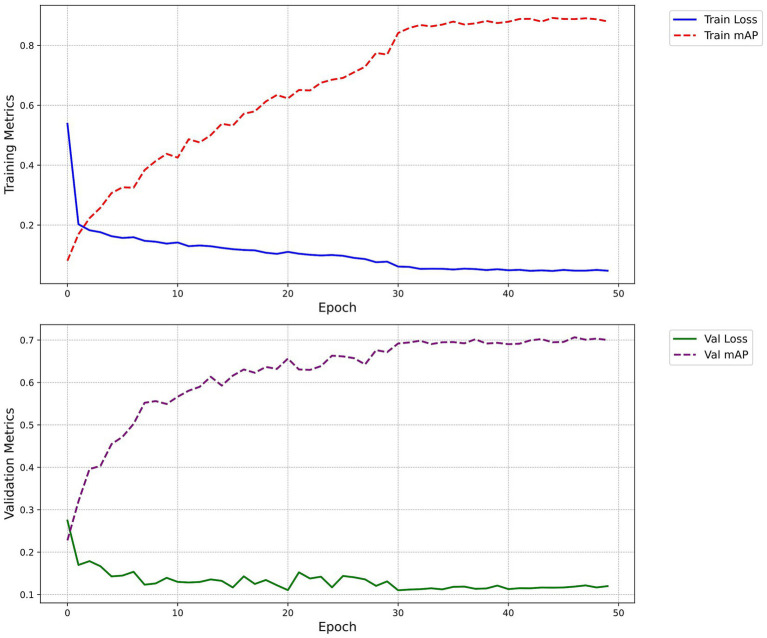
Loss and mAP change curves. The figure demonstrates the progression of the model’s loss and mAP across training epochs, both on the training and validation sets. The blue solid line signifies the train loss, the red dashed line represents the train mAP, the green solid line indicates the Val loss, and the purple dashed line depicts the Val mAP.

Figure 7 illustrates the changes in mAP and loss on the training and validation sets across epochs. The loss curves smoothed out after the 30th epoch, and the mAP peaked and stabilized around the 50th epoch, indicating optimal model performance.

### 4.5 Experimental results

To establish the reliability of our model, we performed experiments on two medical image datasets. In this study, given the exploratory nature of the research and the distinctive characteristics of the data, we have opted not to employ formal statistical analysis. Instead, our focus has been on illustrating and comparing the algorithm’s performance.

#### 4.5.1 Experiments on MuReD2022

The MuReD2022 dataset serves as a crucial component of our experimental evaluation, providing a robust framework to test the efficacy of our Med-DGTN model in multi-label medical image classification. Specifically designed to capture the intricacies of retinal diseases, this dataset offers a valuable resource for both model training and validation. Our experiments on this dataset seek to illustrate how the incorporation of dynamic pathological correlation modeling and low-frequency feature extraction can improve diagnostic accuracy and reliability.

##### 4.5.1.1 Retinal disease dataset MuReD2022

This study primarily used the retinal disease dataset MuReD2022 (27), which integrates images from ARIA, STARE, and RFMiD, totaling 2,208 images. Of these, 1,764 images were utilized as the training set, while 444 images were served as the validation set. The dataset includes 20 distinct labels, such as the “normal” label for healthy retinas and the “Other” label for rare disease conditions. There are variations in image quality and resolution, and each image comprises one or more labels.

##### 4.5.1.2 Experimental results and analysis on MuReD2022

On the MuReD2022 dataset, we compared our Med-DGTN model with classical fundus multi-label classification models, such as Transformer-based models (27). We also concentrated on comparing advanced GCN-based models, including ML-GCN, MGTN (28), and GATN. To ensure fairness, we standardized the CNN module configurations in our comparative analysis. Specifically, ML-GCN used ResNet-101 (18) and DenseNet161, MGTN used ResNeXt50 (28) and DenseNet161, and GATN used ResNeXt-101 (19) and DenseNet161. The experimental results are presented in Table 2.

**Table 2:** Comparisons with SOTA methods on the MuReD2022 dataset.

Methods	mAP	OP	OR	OF1	CP	CR	CF1
C-Tran (27)	68.500	–	–	0.5730	–	–	–
ML-GCN(ResNet-101) (18)	58.101	0.2651	0.6158	0.3706	0.5853	0.5595	0.5721
ML-GCN(DenseNet161)	63.974	0.7140	**0.6768**	0.6949	0.6058	**0.5857**	0.5956
MGTN(ResNeXt50) (28)	57.349	0.7153	0.5548	0.6249	0.5759	0.4346	0.4954
MGTN(DenseNet161)	65.292	0.7099	0.6679	0.6883	0.5969	0.5824	0.5896
GATN(ResNeXt-101) (19)	62.300	0.7282	0.6445	0.6838	0.6343	0.5172	0.5738
GATN(DenseNet161)	68.759	0.7278	**0.6768**	**0.7014**	0.5967	0.5744	0.5853
Ours	**70.654**	**0.7287**	0.6607	0.6930	**0.6386**	0.5756	**0.6055**

In the table, data in bold indicate the best values for their respective metrics, while “–” denotes missing data. Boldface numbers denote the highest performance achieved among all compared models for each evaluation metric.

By examining the data in Table 2, it becomes evident that when DenseNet161 is chosen as the foundational model for the CNN module within the dual-branch structure, it demonstrates a notable advantage in the comprehensive metric, mAP, by 5–8% over other basic models. Under identical conditions, our method consistently achieves the highest mAP value. This is 6.680, 5.362, and 1.895 percentage points higher than ML-GCN, MGTN, and GATN respectively, and is a 2.1 percentage point improvement over C-Tran. Furthermore, our method also excels in six additional indicators (OP, OR, OF1, CP, CR, CF1), obtaining the top performance in OP, CP, and CF1. This suggests that our method not only enhances the extraction of fine-grained features but also optimizes the modeling of label relationships, thus significantly enhancing overall classification performance.

#### 4.5.2 Experimental results on ChestXray14

To further evaluate the effectiveness of our Med-DGTN model in multi-label medical image classification, we conducted experiments on the ChestXray14 dataset. This dataset provides a diverse set of chest X-ray images with multiple disease labels, thereby providing an ideal testing ground for the model’s ability to manage complex multi-label scenarios.

##### 4.5.2.1 ChestXray14 Dataset

In order to further substantiate the feasibility of our method on diverse medical images, we opted for the ChestXray14 dataset (29) provided by the NIH. This dataset comprises of 112,120 frontal-view chest X-ray images, sourced from 32,717 patients. Among these, 86,524 images were utilized for training, while 25,596 were reserved for testing. The dataset encompasses 14 prevalent chest disease labels and a single “No Finding” label. Each image possesses a resolution of 1,024 × 1,024 pixels, and it either bears the “No Finding” label or one or more chest disease labels.

##### 4.5.2.2 Experimental results and analysis on ChestXray14

To ascertain the generalizability of our method in multi-label medical image classification, we executed supplementary experiments on the ChestXray14 dataset. The results, assessed using the AUC metric, were compared with other competitive methods, as displayed in Table 3.

**Table 3:** Comparisons of AUC with SOTA methods on the ChestXray14 dataset.

Disease	Ours	Ref. (29)	Ref. (33)	Ref. (34)	Ref. (35)	Ref. (36)	Ref. (37)
Atelectasis	**0.829**	0.716	0.800	0.781	0.797	0.802	0.823
Consolidation	0.807	0.708	0.800	0.754	0.725	0.796	**0.810**
Infiltration	**0.739**	0.609	0.700	0.702	0.724	0.702	0.731
Pneumothorax	**0.901**	0.806	0.870	0.857	0.869	0.900	0.900
Edema	0.881	0.835	0.880	0.850	0.860	0.883	**0.902**
Emphysema	0.922	0.815	0.910	0.908	**0.933**	0.915	0.921
Fibrosis	0.813	0.769	0.780	0.830	**0.849**	0.825	0.816
Effusion	0.875	0.784	0.870	0.829	0.844	0.874	**0.882**
Pneumonia	**0.781**	0.633	0.670	0.729	0.739	0.715	0.761
Pleural_Thickening	0.755	0.708	0.760	0.778	0.753	0.791	**0.801**
Cardiomegaly	0.907	0.807	0.870	0.880	**0.911**	0.894	0.908
Nodule	0.770	0.671	0.750	0.773	**0.802**	0.768	0.798
Mass	0.851	0.706	0.830	0.834	0.836	0.843	**0.862**
Hernia	**0.945**	0.767	0.770	0.917	0.916	0.943	0.883
Average	0.841	0.738	0.804	0.816	0.826	0.832	**0.842**

The table provides a comparative analysis of our method’s performance with 6 other SOTA methods on the ChestXray14 dataset. The first column delineates the 14 chest disease labels, with the final row’s average representing the cumulative AUC for these 14 labels. Boldface values represent the AUC scores corresponding to the best-performing model across individual disease categories.

Our model outperforms others in Atelectasis, Infiltration, Pneumothorax, Pneumonia, and Hernia, achieving the highest AUC values. Although the average AUC (0.841) is marginally lower than that of CoAtNet (0.842), our method demonstrates a substantial advantage in the intricate Pneumonia label, with a 2% higher AUC. This underscores our method’s capability in identifying complex pathological features, particularly in challenging diseases such as pneumonia.

### 4.6 Ablation studies

To thoroughly evaluate the contributions of individual components in our proposed methodology, we carried out a series of ablation experiments. Specifically, we methodically removed or substituted key components of the model and rigorously assessed the effects of these modifications on model performance. This experimental design aids in elucidating the roles and efficiencies of each component within the model. All ablation experiments were executed on the MuReD2022 dataset.

As illustrated in Table 4, we initially evaluated the complete model with all components intact. Upon replacing the WTDense Block with a standard Dense Block, we noted a 2.694 drop in the mAP metric. This finding underscores the pivotal role of the WTDense Block in augmenting the model’s feature extraction and representation capabilities, particularly in enhancing overall performance and refining feature fusion.

**Table 4:** Ablation study on MuReD2022.

Baseline	WTDenseBlock	Graph Transformer	mAP
√			63.974
√	√		64.700
√		√	67.960
√	√	√	**70.654**

This table provides a comparison of model performance on the MuReD2022, showcasing different combinations of the baseline model with the WTDenseBlock module and the Graph Transformer module. “√” indicates that the corresponding module is included in the model configuration. Boldface values represent the mAP scores achieved by the best-performing configuration in the ablation experiments.

Subsequently, when we dispensed with the Graph Transformer driven dynamic label correlation matrix method for constructing the correlation matrix, the model’s mAP metric experienced a significant decline of 5.954. This outcome further corroborates the importance of this method in facilitating the model’s generation of more informative correlation matrices, especially in enhancing the model’s capacity to comprehend and capture intricate relationships.

Compared to the baseline model ML-GCN (based on DenseNet161), our complete model demonstrated a substantial improvement of 6.68 in mAP. This result exemplifies the synergistic effect of enhancing fine-grained feature extraction in medical images via WTConv and optimizing label topology modeling using the Graph Transformer. Collectively, these enhancements significantly elevate the model’s overall performance.

## 5 Discussion

The Med-DGTN model addresses two core challenges in multi-label medical image classification: modeling dynamic pathological correlations and extracting low-frequency features. The Graph Transformer layer dynamically refines the label dependency graph during training, allowing the model to capture asymmetric and clinically meaningful co-occurrence patterns, which are common in ophthalmic conditions such as age-related macular degeneration, diabetic retinopathy, and glaucoma. This adaptive modeling reflects disease relationships more realistically than static approaches. In addition, the WTDense Block enhances the extraction of low-frequency features through wavelet-based convolution, which is particularly effective for identifying subtle pathological signs—such as drusen, or mild optic disc swelling—that may be underrepresented in training data. These enhancements support more accurate recognition of complex disease presentations in fundus imaging.

Experimental results on the MuReD2022 dataset reveal that Med-DGTN achieves a 70.65% mAP. When compared to other robust models within the CNN-GCN framework using DenseNet as the base CNN, Med-DGTN surpasses ML-GCN, MGTN, and GATN by 6.680, 5.362, and 1.895 percentage points, respectively, and C-Tran by 2.1 percentage points. Med-DGTN also demonstrates superior performance in other metrics such as OP, CP, and CF1. On the ChestXray14 dataset, Med-DGTN excels in five out of fourteen labels. Notably, it exhibits a 2% improvement in the AUC for the intricate Pneumonia label, suggesting that the WTDense Block effectively captures low-frequency pathological features. However, Med-DGTN underperforms compared to SOTA methods on labels like Fibrosis, possibly due to weak associations with other disease labels, which limits the effectiveness of the dynamic adjacency matrix. Ablation studies confirm the importance of each component by demonstrating significant performance declines when replacing or removing modules.

Although the DAME module offers a flexible, data-driven approach to dynamically constructing the label graph through statistical and learned dependencies, its implementation on large-scale datasets introduces practical challenges. First, the computation of the label co-occurrence matrix $M\in {\mathbb{R}}^{C\times C}$ , where C is the number of categories and N is the number of samples, has a complexity of $O(C^{2}\cdot N)$ . While this step is typically performed during preprocessing and does not cause significant overhead in most cases, it can become resource-intensive when the number of labels is large. To address this, the correlation matrix can be computed and stored before training begins. In the graph attention stage, constructing the query, key, and value vectors and computing the scaled dot-product attention further increases the complexity to $O(C^{2}\cdot d)$ , where d is the attention dimension. In practice. For instance, when C=20 (as in the MuReD2022 dataset) and d=64 ,the attention mechanism computes 25,600 weights. When C=60 , this increases to 230,400 weights, which may lead to additional GPU memory usage during training. In practice, computational costs can be mitigated by limiting the number of computed subgraphs, applying threshold-based sparsity constraints, or grouping low-frequency labels together. These strategies allow the model to remain scalable while preserving the richness of inter-label relations.

Despite other researchers working on multi-label classification for MuReD2022 and ChestXray14, differences in evaluation metrics and the lack of code availability make their methods irreproducible (30). Therefore, comparisons are only made with methods that have available code or use the same evaluation metrics.

## 6 Conclusion

Multi-label classification presents significant importance and complex in medical image analysis, as a single image may display multiple disease characteristics. The primary challenges include modeling dynamic pathological correlations and extracting low-frequency pathological features. This paper introduces the Med-DGTN model, which employs a CNN-GCN cross-modal alignment strategy to achieve a deep coupling of image features and label semantics. The Graph Transformer layer effectively captures dependencies between diseases, while the WTDense Block module enhances low-frequency feature extraction through wavelet decomposition. Experimental results show that the Med-DGTN model achieves outstanding performance on the MuReD2022 and ChestXray14 datasets. These results demonstrate the model’s potential to assist in real-world clinical settings by improving the accuracy and comprehensiveness of automated disease screening. For instance, by accurately identifying co-existing pathologies in a single scan, Med-DGTN can support radiologists in making more informed and efficient diagnostic decisions, particularly in high-throughput environments. Future research may explore alternative graph-based techniques, such as GraphSAGE (31) and Node2Vec (32), to further enhance the modeling of disease relationships. In addition, incorporating domain-specific medical word embeddings may offer improved semantic representations of disease labels compared to general-purpose embeddings. These approaches can provide more flexible and scalable representations of label dependencies in large-scale graphs, potentially improving the quality of the dynamically constructed adjacency matrix and boosting model performance on complex medical datasets.

## Data Availability

The original contributions presented in the study are included in the article/supplementary material, further inquiries can be directed to the corresponding authors.
